# TLR2 ligand-synthetic long peptide conjugates effectively stimulate tumor-draining lymph node T cells of cervical cancer patients

**DOI:** 10.18632/oncotarget.11512

**Published:** 2016-08-23

**Authors:** Gijs G. Zom, Marij J.P. Welters, Nikki M. Loof, Renske Goedemans, Sinéad Lougheed, Rob R.P.M. Valentijn, Maarten L. Zandvliet, Nico J. Meeuwenoord, Cornelis J.M. Melief, Tanja D. de Gruijl, Gijsbert A. Van der Marel, Dmitri V. Filippov, Ferry Ossendorp, Sjoerd H. Van der Burg

**Affiliations:** ^1^ Department of Immunohematology and Blood Transfusion, Section Tumorimmunology, Leiden University Medical Center, Leiden, The Netherlands; ^2^ Department of Clinical Oncology, Leiden University Medical Center, Leiden, The Netherlands; ^3^ Department of Medical Oncology, VU University Medical Center, Amsterdam, The Netherlands; ^4^ Department of Clinical Pharmacy and Toxicology, Leiden University Medical Center, Leiden, The Netherlands; ^5^ Leiden Institute of Chemistry, University of Leiden, Leiden, The Netherlands; ^6^ ISA Pharmaceuticals BV, Leiden, The Netherlands

**Keywords:** Toll-like receptor ligand, conjugate, synthetic long peptide, cancer vaccine, cervical cancer

## Abstract

The potency of human papillomavirus type 16 (HPV16)-encoded synthetic long peptides (SLP), conjugated to an optimized Toll-like receptor 2 ligand (TLR2-L), was assessed in *ex vivo* activation of HPV16^+^ cancer patient-derived T cells. Two highly immunogenic SLP sequences derived from the oncogenic E6 protein of HPV16 were selected and conjugated to a Pam3CSK4-based TLR2-L under GMP conditions. Both conjugates were able to mature human DCs *in vitro* and to activate human skin-derived antigen-presenting cells (APCs) upon intradermal injection in an *ex vivo* skin model, associated with induction of a favorable chemokine profile to attract and activate T cells. The conjugated SLPs were efficiently processed by APCs, since HPV16-specific CD4^+^ and CD8^+^ T-cell clones isolated from HPV16+ cervical tumors proliferated in response to both conjugates. The TLR2-L SLP conjugates significantly enhanced *ex vivo* T helper type 1 T-cell activation in cell suspensions obtained from tumor-draining lymph nodes (LN) resected during hysterectomy of HPV16^+^ cervical cancer patients. These results show that TLR2-L SLP conjugates can activate circulating or LN-derived tumor-specific T cells, a promising outcome for studying these two conjugates in a phase I/II clinical safety and immunogenicity trial.

## INTRODUCTION

Cancer immunotherapy aims to instruct the patient's immune system to eradicate tumors. Synthetic long peptide (SLP)-based vaccines constitute such a therapy to augment pre-existing, but often weak or suppressed, T cell responses in cancer patients. Synthetic long peptides (SLPs) are processing-dependent linear amino acid sequences harboring multiple potential CD4^+^ and CD8^+^ T cell epitopes that are expressed by tumor cells. We have previously reported the immunological advantages of these chemically well-defined long peptide vaccines, that are readily produced under GMP [1, 2].

In-depth analysis of T cell responses in the tumor and in tumor-draining lymph nodes (LN) of HPV16^+^ cervical cancer patients revealed that T cells directed against multiple epitopes of the HPV16 E6 and E7 oncoproteins are present, not as fully activated T cells, but poised for action [3]. Indeed, reactivation of these T cells can readily be achieved by vaccinating HPV16^+^ cervical cancer patients with an SLP vaccine consisting of overlapping SLPs covering HPV16 E6 and E7 [4, 5]. Clinical benefit of SLP vaccination was established in patients with HPV16-induced premalignant lesions. At 12 months of follow-up, clinical responses were observed in 52 to 79% of patients with high-grade vulvar and vaginal intraepithelial neoplasia (VIN/VaIN), as recently reported in two independent clinical trials studying the efficacy of the HPV16 SLP vaccine [6, 7]. Immune monitoring showed that SLP vaccination induced HPV16-specific T cell responses in all patients and that clinical efficacy correlated with a strong IFNγ-associated T cell response in both trials.

SLP vaccination induces strong CD4^+^ T cell responses. The CD8^+^ T cell responses are weaker and more difficult to detect, suggesting that these cells require efficient boosting, e.g. by the addition of an effective adjuvant [8, 9]. TLR-ligands are generally well-known for their capacity to boost T cell responses *in vivo* [10–12]. A study by De Vos van Steenwijk *et al* showed that *ex vivo* stimulation of cervical cancer-infiltrated and tumor draining-LN T cells using specific peptides mixed with a TLR agonist resulted in a strongly enhanced IFNγ-expression [3]. Although the application of imiquimod at the vaccination site in our recent HPV16 SLP clinical trial did not improve T cell responses and clinical outcome, the application of TLR-ligands in cancer vaccines remains promising when provided in an optimal setting [13].

Covalent conjugation of a TLR2-L to SLPs constitutes an even more sophisticated approach to improve the current HPV16 SLP vaccine [14–17]. In murine models, we have shown that TLR2-L SLP conjugates are targeted *in vivo* to antigen-presenting cells (APCs) expressing TLR2 and thereby improve antigen uptake, simultaneously maturing these APCs [14, 18]. This dual effect on the APCs resulted in strongly increased T cell priming *in vivo*, as well as enhanced induction of anti-tumor immunity [15]. Importantly, the improved efficacy of TLR2-L SLP conjugates allows a significant reduction in dose, thereby potentially reducing the risk of injection site reactions [6, 7, 19].

Upon injection, vaccine components readily gain access to several local APC types, which can subsequently ingest the antigen and become activated by adjuvant-formulated vaccines. Intradermal injection of HPV16 SLPs without adjuvants was shown to induce circulating and skin-infiltrating HPV16-specific CD4^+^ and CD8^+^ T cells [20]. Adjuvants have the capacity to modulate local skin-associated APCs to induce either a Th1- or Th2-type response by upregulation of co-stimulatory activation markers and expression of cytokines and chemokines [21]. By conjugating a TLR2-L to an SLP, we have developed a synthetically well-defined vaccine that has the potency to cause local innate immune activation, antigen-targeting to DC and DC activation, together leading to efficient T cell activation.

The present study describes the development, immunological potency and capacity to prime and boost cancer patient-derived T cells *ex vivo* of TLR2-L SLP conjugates that are currently tested in a clinical trial. We show that the TLR2-L SLP conjugates induce significant activation of HPV16-specific CD8^+^ and CD4^+^ T cells and robust expression of IFNγ and/or IL-2 by *ex vivo* stimulated tumor-draining LN-derived T cells of cervical cancer patients.

## RESULTS

### Synthetic long peptide amino acid sequences 71-95 and 127-158 of the HPV16 E6 protein represent highly immunogenic regions

**Figure 1:**
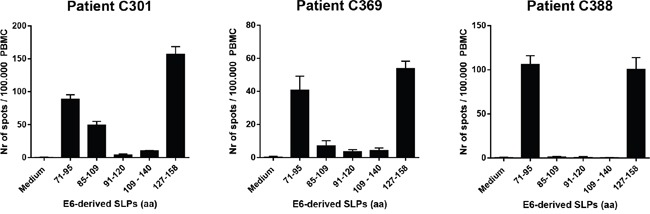
Synthetic long peptides (SLPs) with amino acid (aa) sequences E6_71-95_ and E6_127-158_ induce strong IFNγ-responses in PBMC of cervical cancer patients PBMC of three cervical cancer patients (C301, C369 and C388) were tested. Data represent average spot counts of quadruplicate wells with standard deviations in the IFNγ ELISpot assay after peptide stimulation of PBMC for 4 days. Horizontal axis: amino acid sequences of HPV16 E6-derived peptides used to stimulate PBMC.

The current HPV16 SLP vaccine consists of thirteen peptides covering the entire amino acid sequences of the HPV16 E6 and E7 oncogenic proteins. For proof of principle, we decided to select the two most immunogenic regions of the HPV16 E6 antigen, based on the spontaneous immune reactivity in healthy immune donors without evidence of virus infection. Two SLPs representing these two regions were used for conjugation to the optimized TLR2-ligand Amplivant™ (AV), followed by pre-clinical testing of their functionality. Based on the spontaneous immune reactivity in the protected healthy donors the C-terminal half of the HPV16 E6 protein constitutes the most immunogenic region (Supplementary Figure S1A) [22, 23]. In agreement with these results, tumor-draining LN cells derived from 9 cervical cancer patients (De Vos van Steenwijk et al. [3] and unpublished data) predominantly responded to epitopes present in the center and C-terminal region of E6 (Supplementary Figure S1B). Subsequently, we analyzed three cervical cancer patients who responded to the C-terminal half of E6 after vaccination with the HPV16 SLP vaccine [4], to identify which peptides contained in the vaccine were the most immunogenic. All three tested PBMC samples showed a strong response against epitopes within the HPV16 E6 71-95 and 127-158 SLPs (Figure 1A). Analysis of these two peptides using the MHC algorithm databases IEDB and SYFPEITHI revealed that both SLP sequences harbor a multitude of potential HLA class I- and II-binding epitopes for frequent HLA-alleles in The Netherlands (Table 1). Based on these combined findings, the HPV16 E6 71-95 (SLP_71-95_) and 127-158 (SLP_127-158_) SLPs were selected for conjugation to a TLR2-L.

**Table 1:** Number of predicted HLA-binding peptides within the indicated amino acid sequences of HPV16 E6, using two online epitope prediction algorithms

	E6 71-95	E6 127-158
HLA-A*01:01	4	1
HLA-A*02:01	3	2
HLA-A*03:01	3	4
HLA-A*11:01	3	4
HLA-A*24:02	8	2
HLA-A*26:01	4	0
HLA-B*07:02	3	2
HLA-B*08:01	2	4
HLA-B*15:01	2	0
HLA-B*35:01	4	0
HLA-B*40:01	2	1
HLA-B*44:02	3	1
HLA-B*51:01	2	1
HLA-DR1*B01	2	3
HLA-DR1*B03	2	1
HLA-DR1*B04	1	2
HLA-DR1*B07	2	3
HLA-DR1*B11	1	1
HLA-DR1*B15	3	3

### HPV16 E6-derived SLPs conjugated to TLR2-L mature human dendritic cells and induce chemokine responses

**Figure 2:**
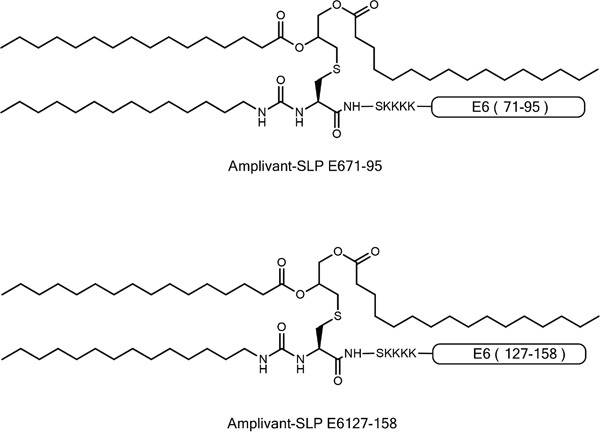
Representation of AV-SLP conjugates with HPV16 E6-derived amino acid sequences 71-95 and 127-158

**Figure 3:**
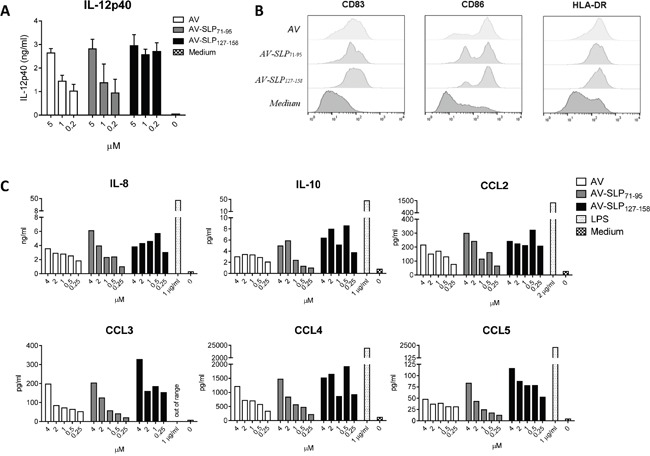
Conjugates of TLR2-L and HPV16-derived SLPs mature human DCs **A.** Production of IL-12p40 as measured in supernatant of human monocyte-derived DCs (moDCs) from 1 donor stimulated with AV only (AV) or AV-SLP conjugates. Error bars represent standard deviation of triplicate wells. Data are representative for 5 similar experiments. **B.** Expression of co-stimulatory molecules CD83 and CD86 and HLA-DR on human moDCs after stimulation with 2 μM of compounds as in (A). **C.** Concentrations of IL-8, IL-10, MCP-1, CCL3, CCL4 and CCL5 in supernatant of human moDC stimulated with titrating doses of compounds, as measured by Luminex assay. TLR4-ligand LPS was added as a positive control. Data shown are representative of 5 similar experiments.

We generated conjugates of the two selected HPV16 E6-derived SLPs by in-line peptide synthesis and final attachment to AV as described by Khan *et al* (Figure 2 and Supplementary Figure S2) [14]. AV is an improved Pam3CSK4-based TLR2-binding ligand, generated by modifying one of the three lipid tails of Pam3CSK4 to enhance binding of AV to its receptor. AV was shown to induce stronger maturation of murine DCs than Pam3CSK4 [24]. AV and AV-SLP conjugates induced activation of TLR2-expressing HEK293 cells but not wild-type HEK293 cells (Supplementary Figure S3), showing that AV and the AV-SLP conjugates specifically activate cells through TLR2. To determine whether AV retains its potency to induce DC maturation and activation after conjugation to long peptides, monocyte-derived DCs (moDC) were incubated with each of the conjugates or AV alone. After co-culture for 24 hours, the concentration of IL-12p40 in the supernatant was measured by ELISA. Both AV-SLP conjugates and AV alone triggered IL-12p40 production (Figure 3A) and the expression of the maturation markers CD83, CD86 and HLA-DR (Figure 3B). Despite reports on the occurrence of polymorphisms in the TLR2 gene locus [25–27], we have reproducibly observed maturation of human moDCs derived from PBMC of all tested healthy donors (n=20, data not shown). This shows that AV can target and activate relevant APCs irrespective of potential TLR polymorphisms in the patient population. AV and AV-SLP conjugates also triggered the production of chemokines by DCs. Incubation of human moDCs with AV or with the conjugates induced the secretion of IL-8, IL-10, MCP-1, CCL3, CCL4 and CCL5 (Figure 3C), albeit not to the same levels as seen after incubation of DCs with the strong TLR4 agonist LPS.

### Induction of a favorable, T cell attracting, chemokine profile by TLR2-L SLP conjugates in human skin explants

**Figure 4:**
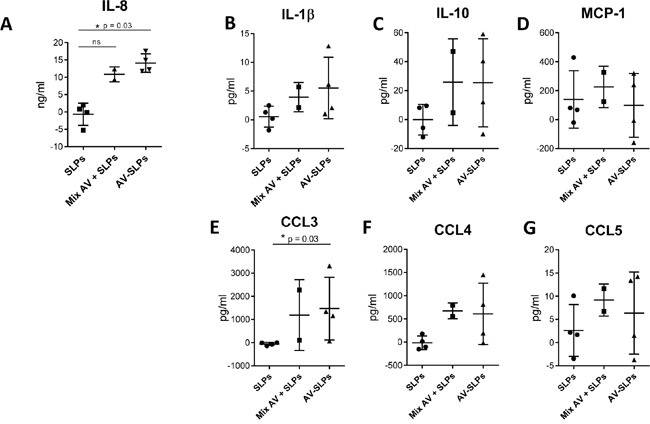
Conditioning of the skin microenvironment by AV-SLP conjugates at the vaccination site leads to the release of pro-inflammatory cytokines and chemokines Cytokine and chemokine production by human skin-migratory cells after intradermal injection of a mix of HPV16 E6-derived SLP_71-95_ and SLP_127-158_, a mixture of AV with both SLPs or a mix of both AV-SLP conjugates in human skin explants (n=4). **A.** IL-8 concentration in supernatant, as measured by ELISA. **B-G.** IL-1β, IL-10, MCP-1, CCL3, CCL4 and CCL5 concentration in supernatant as measured by Luminex assay. Background cytokine concentration in saline condition was subtracted from the test conditions of each donor skin. Error bars represent standard deviations of the 4 tested skin explants. Significant differences (* p < 0.05) determined by Mann Whitney U test.

Intradermal vaccination of AV-SLPs offers an attractive alternative for SLP vaccination [20, 28] to avoid side effects by Montanide emulsions [6, 7, 19]. We studied the potency of the AV-SLP conjugates in activating skin-associated cells upon intradermal injection by making use of skin explants from four different healthy donors (Figure 4) [29]. Directly following intradermal injection, a 6 mm diameter biopsy was taken from the area of the injection site. Skin-migratory cells were allowed to migrate for 48 hours from the biopsy into culture medium-filled wells. Flow cytometry analysis revealed that on average approximately 75% of migrated cells were CD11c^+^ (ranging from 50% to 90%). The composition of CD1a^+^ and CD14^+^ migratory DC subsets was not significantly changed after injection of the AV-SLPs (Supplementary Figure S4B). Chemokine levels in the skin supernatants were measured using ELISA (IL-8) or Luminex. The concentrations of IL-1β, CCL4, IL-10 and in particular IL-8 and CCL3 were increased in all biopsies injected with AV-SLPs compared to SLP-injected skin biopsies. This confirms our *in vitro* data on AV-stimulation of moDC and demonstrates that intradermal injection of the AV-SLPs or mixtures of AV and SLP stimulates stronger local inflammation than injection of free SLPs. It also shows that AV retains its adjuvant potency upon conjugation to SLPs after injection in human skin.

### AV-SLPs induce T cell proliferation of HPV16-specific CD8 and CD4 T cell clones

**Figure 5:**
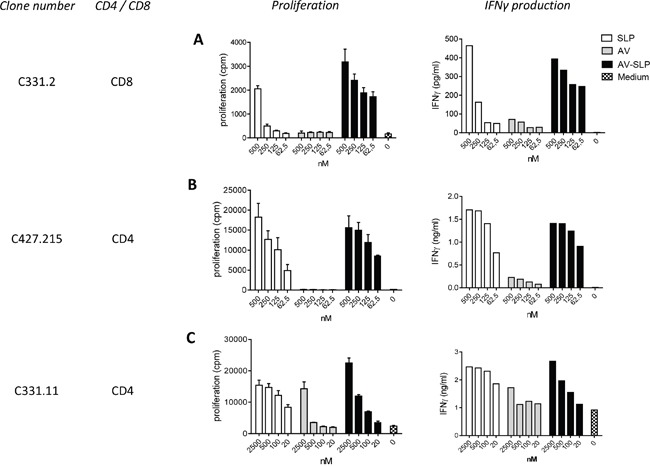
Enhanced CD8^+^ and similar CD4^+^ T cell clone proliferation induced by AV-SLP conjugates as compared to free SLP Proliferation and IFNγ production of **A.** CD8^+^ T cell clone C331.2 and **B.** CD4^+^ T cell clone C427.215 after co-culture with HLA-matched moDCs loaded with SLP_127-158_. **C.** Proliferation and IFNγ production of CD4^+^ T cell clone C331.11 after co-culture with HLA-matched moDCs loaded with SLP_71-95_. Error bars represent standard deviation of triplicate wells.

Next, we determined whether the conjugated peptides are processed by APCs and whether the embedded MHC class I and II epitopes are properly presented. Three T cell clones responding to the selected peptides were isolated from cervical cancer infiltrating lymphocytes of two patients [30]. From these clones, CD8^+^ T cell clone C331.2 and CD4^+^ T cell clone C331.11 are specific for an epitope in SLP_127-158_, while CD4^+^ T cell clone C427.215 is specific for an epitope in SLP_71-95_. HLA-matched moDCs were pulsed for 24 hours with the AV-SLP conjugates, free AV or SLP alone and subsequently used to stimulate the T cell clones. The activation of CD8^+^ T cell clone C331.2, measured by proliferation and IFNγ production, was much stronger to DCs loaded with AV-SLP_127-158_ than with SLP only (Figure 5A). Both CD4^+^ T cell clones responded equally well to conjugated and non-conjugated SLP (Figure 5B, 5C). The non-specific proliferation of clone C331.11 when co-cultured with DCs stimulated with the highest dose of AV probably is due to the high maturation status of the stimulated DCs. Overall, these data show that the E6 SLPs conjugated to AV are taken up and processed efficiently by DCs to generate and present T cell epitopes in both MHC class I and II molecules. Remarkably, the activation of the CD8^+^ T cell clone was improved upon conjugation of the SLP to AV suggesting a more efficient processing and presentation of AV-SLP conjugates than the free SLPs into MHC class I.

### Cancer patient-derived lymph node T cells produce more Th1-type cytokines upon stimulation with AV-SLP conjugates

**Figure 6:**
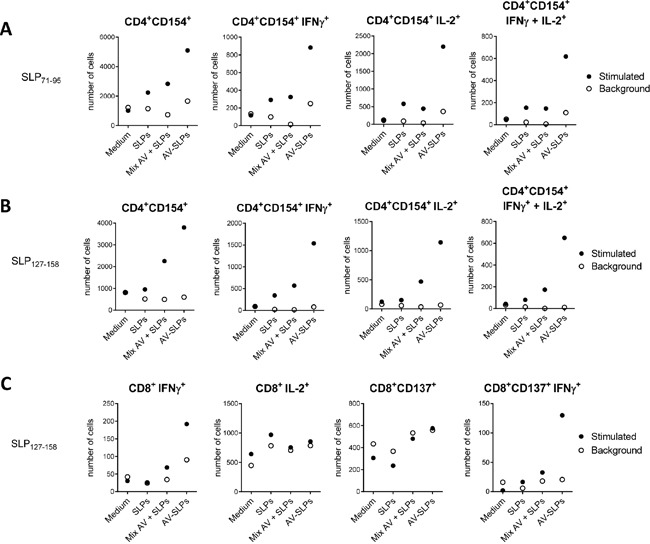
AV-SLP conjugates enhance IL-2 production by CD4^+^ LN T cells and IFNγ production by CD8^+^ LN T cells isolated from patient C427 **A.** Total number of cells in culture expressing CD154^+^ on CD4^+^ T cells specific for an epitope within SLP_71-95_, and total number of cells in culture expressing intracellular levels of IFNγ and IL-2 in the indicated populations. Flow cytometry was performed after a 3-week co-culture of monocytes loaded with indicated compounds and LN-derived T cells. Background represents marker expression after re-stimulation of T cells one day prior to intracellular cytokine staining. SLPs: equimolar mix of HPV16 SLP_71-95_ and SLP_127-158_; AV-SLPs: equimolar mix of AV-SLP_71-95_ and AV-SLP_127-158_ **B.** Idem for CD4^+^ T cells specific for an epitope within E6_127-158_. **C.** Idem for CD8^+^ T cells specific for an epitope within E6_127-158_.

We have previously observed that tumor-draining LN-derived T cells of a cervical cancer patient were able to respond against epitopes in both selected E6 SLPs [3]. Here we analyzed the *ex vivo* activation of these tumor-draining LN cells after stimulation with the AV-SLP conjugates. Autologous monocytes were loaded with the individual AV-SLP conjugates (AV-SLP_71-95_ or AV-SLP_127-158_), a mixture of free SLPs and AV or with free SLP for 24 hours and then used as APCs. A higher number of activated CD4^+^ T cells expressing CD154 (CD40L) was observed in the LN culture stimulated with AV-SLP as compared to the controls (Figure 6A and 6B). Also the numbers of activated IFNγ and IL-2 single and double producing T cells were higher when SLPs were conjugated to AV (Figure 6A and 6B). In addition, CD8^+^ T cell reactivity was improved as the number of IFNγ-producing E6_127-158_–specific CD8^+^ T cells was also higher after stimulation with the AV-SLP conjugate (Figure 6C). These data show that T cells present in the tumor-draining LN can be more efficiently activated by stimulation with AV-SLP conjugates than with the free SLPs or mixtures of free SLPs and AV.

### T cell responses frequently observed in cervical cancer patient lymph node T cell cultures upon *in vitro* stimulation

Because we had identified SLPs that were recognized by a high proportion of cervical cancer patients and healthy individuals, we here tested the capacity of the AV-SLP conjugates to stimulate tumor-draining LN cells from randomly selected HPV16^+^ cervical cancer patients. LN mononuclear cells from six randomly chosen HPV16^+^ cervical cancer patients were stimulated *ex vivo* with a mix of both AV-SLP conjugates, a mix of both free SLPs or medium as a control. Activation of HPV16 E6-specific T cells was measured by cytometric bead array (CBA) on supernatant collected after 7 or 8 days of culture. The detected concentrations of IFNγ, IL-5 and IL-10 were generally above the lower detection limits of the assay and ranged from 12.9 pg/ml – 17.7 ng/ml (IFNγ), 2.5 – 325.2 pg/ml (IL-5), 4.5 – 172.4 pg/ml (IL-10). TNFα was not detected in the cultures of C822 and C830, and ranged from 7.4 – 77.5 pg/ml in the other cultures. The LN cultures of two out of six cancer patients showed an IFNγ response after stimulation with the AV-SLP conjugates (C879, C896; Table 2). Both of the responding patients also showed induction of IL-5 upon stimulation with the AV-SLP conjugates. The LN culture derived from patient C822 responded to stimulation with free SLPs by producing IL-5 only. IL-10 and the Th2-associated cytokine IL-4 were also measured, but no significant levels could be detected in any of the supernatants. In addition, despite the lack of detectable cytokines in CBA, we could observe an increase in IFNγ-production in LN cell cultures obtained from patient C972 by ICS after 12 days of culture (Supplementary Figure S5). A suboptimal timing of measurement resulting in still high background activation levels of cells following the initial stimulation precluded the detection of responses using ICS in the other patient lymph node cell cultures. Overall, these findings show that in 3 (C879, C896 and C972) out of 6 tested HPV16^+^ cancer patients, LN T cells effectively responded to the two combined AV-SLP conjugates.

**Table 2:** Cytokine responses measured in the supernatant of cervical cancer patient lymph node cell cultures

		C822	C830	C879	C894	C896	C972
*SLPs*							
	IFNγ	< 1	< 1	1.04	< 1	1.05	1.35
	TNFα	< 1	< 1	1.14	< 1	< 1	1.02
	IL-5	**4.11**	< 1	1.51	< 1	< 1	< 1
	IL-10	< 1	1.59	< 1	< 1	< 1	1.14
*AV-SLPs*							
	IFNγ	< 1	< 1	**5.98**	< 1	**5.71**	< 1
	TNFα	< 1	< 1	1.81	< 1	2.75	< 1
	IL-5	< 1	1.49	**12.09**	1.55	**5.49**	< 1
	IL-10	< 1	1.55	< 1	< 1	2.89	< 1

## DISCUSSION

This study shows that the conjugation of SLPs to an optimized TLR2-L results in an enhanced potency to specifically activate cancer patient-derived T cells. We selected the two most immunogenic SLPs from the thirteen SLPs of a potential therapeutic HPV16 clinical vaccine for conjugation to a defined TLR2-L, designated Amplivant™ (AV). We showed that these AV-conjugated SLPs induce the activation and maturation of human DCs as well as promote local inflammation when injected into the human skin. The conjugates were processed efficiently, leading to a markedly improved activation of cloned tumor-infiltrating CD8^+^ T cells. In addition, the AV-SLP conjugates effectively stimulated Th1-type cytokine CD4^+^ and/or CD8^+^ T cell-responses in tumor-draining LN-derived T cells. Moreover, the AV-SLP conjugates could induce a specific IFNγ^+^ response in three out of six LN cell cultures derived from randomly selected HPV16^+^ cancer patients whereas free SLPs were able to do so in one out of six cultures.

The two HPV16 E6-derived SLPs were selected by analyzing T cell responses against HPV16 E6 and E7 as measured in healthy donors and lymph node material of cervical cancer patients. More T cell responses in cervical cancer patients are directed against the E6 than the E7 protein, as described in Kenter *et al* and Welters *et al* [6, 8]. By analyzing T cell responses in previous studies, it was estimated that at least half of all HPV16-positive individuals could mount a response against these two peptides. In addition to immunogenicity, logistic factors such as feasibility of chemical synthesis, solubility and formulation made us decide to select these two SLPs for conjugation. In this study we did not experience any relevant difficulties in synthesis and solubility of these two conjugates, not even when produced at a larger scale under GMP conditions (unpublished observations).

By choosing a TLR2-L for conjugation, we do not only aim to target the SLPs *in vivo* specifically to APCs that express TLR2 extracellularly, but also choose for a well-defined ligand that can be generated synthetically and may be modified for optimal efficacy. Moreover, it was previously shown that the TLR2-L Pam3CSK4 improved the IFNγ response of tumor-infiltrating lymphocytes (TILs) after specific stimulation and had a slight advantage over Poly(I:C) and LPS in these assays [3]. We further aimed to optimize triggering of TLR2 by modifying Pam3CSK4, and generated AV in this manner [24].

Conjugation of AV to SLP sequences is not detrimental for its biological activity as shown by these two conjugates as well as with several other peptide sequences (our unpublished data). We observed a relative higher potency in DC maturation and TLR2-transfected HEK293 cell activation by the AV-SLP_127-158_ than the AV-SLP_71-95_ conjugate. Apparently, the physical and chemical properties of individual peptides can influence the biological activity of AV.

Upon intradermal vaccination, local skin-associated APCs play an important role in the onset of the T cell response. To assess the effects of AV-SLP conjugates on these APCs, we made use of *ex vivo* human skin explant experiments in which we observed significantly enhanced concentrations of IL-8 and CCL3 upon stimulation with the AV-SLP conjugates. A similar trend was clearly observed for IL-1β, IL-10 and CCL4. Of note, these current observations are in line with our previous observation that active TLR2 agonists impacted cytokine release rather than DC phenotype in this model system [21]. Importantly, the chemokine CCL3 can be highly relevant in a vaccination setting as it can recruit CCR1-, CCR3- and CCR5-expressing leukocytes, such as monocytes, NK cells, T cells and DCs. In addition, CCL3 has been associated with Th1-skewing in mice [33, 34]. Our recent *in vivo* analysis in tumor-bearing mice suggests that TLR2-L SLP conjugate vaccination positively impacts the Th1/Th2 balance as we see a strong CTL induction and efficient antitumor immunity [15]. The relevance of IL-10 production, as induced by AV in both DCs and skin-migrated cells, is ambiguous as this cytokine has been described both as a pro- and anti-inflammatory cytokine [35, 36].

The efficiency of peptide processing and presentation and subsequent T cell activation was studied using HPV16-specific T cell clones that recognize epitopes within the conjugated SLPs. These *in vitro* antigen presentation experiments showed that CD8^+^ T cells were activated significantly better by conjugates than by equimolar amounts of SLP or AV. Also CD4^+^ T cells were activated by conjugates *in vitro* although CD4^+^ T cell stimulation efficacy was similar by the corresponding free peptides. The latter finding has also been observed using mouse CD4^+^ T cells *in vitro* in our previous study [15]. This is most likely explained by differences in MHC class I and class II antigen processing pathways in DCs. However, our *in vivo* vaccination studies clearly show strong dependence on TLR2-L conjugation also for CD4^+^ T cell response induction [15], suggesting that the SLP-targeting effect of the TLR2-L to DCs is highly important for both CD4^+^ and CD8^+^ T cell activation *in vivo*. Therefore, covalent TLR2-L conjugation of T helper epitope-containing SLP is preferred above mixtures of these molecules since *in vivo* targeting and concomitant DC maturation are key in effective T cell priming and activation [18], not only by the induced expression of co-stimulatory molecules and relevant cytokines but also by storage of the targeted antigen in specialized compartments of dendritic cells to sustain antigen presentation *in vivo* [37]. Importantly, the targeting properties of AV-SLP conjugates allow a major dose reduction as compared to free SLPs. The current study presents *in vitro* data in which the targeting effect does not play a role. Therefore, the differences between free SLP and AV-conjugated SLP could rather be explained by enhanced uptake of the SLP, the co-stimulation provided to T cells by the matured APCs and the sustained peptide presentation.

Our data suggest that AV-SLP conjugates are more efficient at inducing a T cell response in the LN than free SLP. The availability of tumor-draining LN cells allowed us to analyze relevant T cell populations that were in close proximity to the tumor. We observed a clear increase of Th1-type cytokine production in these LN-derived specific CD4^+^ and CD8^+^ T cells. This indicates that T cells present in a potentially immunosuppressed tumor-draining area are likely to be poised for action in cancer patients [3], as they are still able to become activated and express a potentially beneficial Th1-phenotype. Also the observation that 3 out of 6 LN cultures with unknown reactivity responded by IFNγ production to AV-SLP conjugates and only 1 out of 6 cultures to free SLP suggests that otherwise undetectable HPV16-specific T cells can be efficiently stimulated by AV-conjugated SLPs. AV-SLP conjugates also appear to be able to induce Th2-type cytokines. The production of IL-5 coinciding with IFNγ has also been observed before both in healthy donors, who have successfully cleared the virus [22] and upon SLP vaccination in previous clinical studies, which was associated with clinical responses [3, 4, 19, 38]. It is therefore unlikely that this negatively impacts the induction of effective antitumor immunity.

The safety and immunological properties of the AV-SLP conjugates are currently being examined in a phase I/II clinical trial in HPV16^+^ cancer patients (EudraCT: 2014-000658-12). Overall, this study provides evidence for the superiority of AV-SLP conjugates over free SLP as a vaccination modality to induce cancer patient-derived T cell responses.

## MATERIALS AND METHODS

### Synthesis of AV-SLP conjugates

Amplivant™ (AV) was generated as described before [24], and conjugated to the N-terminus of HPV16 E6-derived SLPs amino acids 71-95 and 127-158 according to protocols described in Khan et al. [14]. UV-spectrum analysis of the generated conjugates shows a high purity of the syntheses (98.7% for AV-SLP_71-95_ and 99.3% for AV-SLP_127-158_; S2).

### Patient material

Patient material was derived from women presenting with histologically proven cervical neoplasia at the department of Gynaecology of the Leiden University Medical Center and Haga Teaching Hospital, The Hague. These patients were enrolled in the CIRCLE study, which investigates cellular immunity against HPV16-positive cervical lesions after providing informed consent.

### MHC class I and II epitope prediction

Epitope predictions in Table 1 are based on predictions from two online MHC class I and II binding algorithms (IEDB: http://www.iedb.org/ and SYFPEITHI: http://www.syfpeithi.de/). For IEDB, a peptide with a predicted EC_50_ below 8 nM was considered a strong MHC binder. For SYFPEITHI, peptides scoring 15 or higher were considered strong MHC binders.

### ELISpot

IFNγ ELISpot was performed as described previously [4, 5, 31]. In short, PBMC samples of three cervical cancer patients were stimulated at a density of 2 × 10^6^ cells/ml for 4 days in IMDM medium supplemented with 10% human AB serum (Life Technologies) in the presence of 5 μg/ml of indicated peptides. Next, the stimulated PBMC were transferred to an anti-IFNγ-coated (Mabtech) Multiscreen 96-wells plate (Millipore), seeded in quadruplicates at a density of 5 × 10^5^/well. After 24 hours of incubation, the Multiscreen plate was developed according to the manufacturer's instructions (Mabtech). IFNγ-positive spots were counted on a BioSys 5000 video-imaging system. Specific spots were calculated by subtracting the mean value + 2xSD of the medium control wells from the experimental wells. Samples were considered positive when at least 10 spots were measured and the number of spots exceeded the mean number of spots of medium wells by at least 2-fold.

### Monocyte-derived dendritic cell maturation and HEK293 cell activation

Peripheral blood mononuclear cells (PBMC) from healthy donors (Sanquin) were isolated by centrifugation over a Ficoll gradient. Using magnetic CD14 Microbeads (Miltenyi), the CD14^+^ fraction was isolated from these PBMC. The CD14^+^ cells were cultured for 5 days in the presence of 800 U/ml human GM-CSF (Peprotech) and 500 U/ml human IL-4 (Peprotech). On day 5, the monocyte-derived DC (moDC; CD11c^+^ CD1a^+^ CD14^−^ HLA-DR^lo^) were stimulated with the indicated reagents for 48 hours. The concentration of IL-12p40 in the supernatant after 24 hours was measured by ELISA (BioLegend). After 48 hours, the moDC were stained with fluorescently labeled antibodies targeted to CD83, CD86 and HLA-DR (eBioscience), and analyzed by flow cytometry (BD).

WT and TLR2-expressing HEK293 cells (Invivogen) were cultured in IMDM supplemented with 100 IU/ml penicillin/streptomycin (Gibco), 2 mM L-glutamin (Gibco) and 8% fetal calf serum (FCS; PAA Laboratories). TLR2-HEK293 cells were cultured in the presence of 10 μg/ml of the selective antibiotic blasticidin. In a 96 wells plate, 20,000 HEK293 or TLR2-HEK293 cells were seeded per well and stimulated with titrating doses of the indicated compounds in duplicates. After 24 hours of incubation at 37°C, supernatant was taken from all wells and the concentration of IL-8 was measured by ELISA (BioLegend).

### Detection of cytokine and chemokine concentrations

The concentration of single cytokines was detected using standard sandwich ELISA assays (IL-8, IL-12p40, BioLegend; IFNγ, Sanquin), according to the manufacturer's instructions. To study the production of multiple cytokines and chemokines by moDCs or skin-associated cell populations in one sample, cell culture supernatants were subjected to Luminex analysis. For this purpose, a custom panel was set up consisting of reagents detecting the indicated cytokines and chemokines. A Bioplex suspension array system (Bio-Rad) was used to measure cytokine and chemokine concentrations. The analysis was performed according to the manufacturer's instructions (Bio-Rad). Data were analyzed using Bioplex Manager software.

### Activation of skin explant-derived cells

Human skin explants were derived from patients undergoing plastic surgery after giving informed consent, as described earlier [29]. After intradermal injection of 10 μl of the indicated reagents (4 nmole/injection), a biopsy with 6 mm diameter was taken of the injection site. The biopsies (10 for each reagent) were placed in a well to float freely with their epidermal side up on IMDM medium containing 10% FCS. The biopsies were discarded after 48 hours and the pooled medium was analyzed for the presence of an array of chemokines and cytokines by Luminex technology (Bio-Rad) or ELISA (Sanquin). Flow cytometry was performed on a FACS Calibur (BD Biosciences) after staining migrated cells with fluorescent antibodies (BD Biosciences). An example of our gating strategy is presented in Supplementary Figure S4A.

### Proliferation of cancer patient derived T cell clones

The T cell clones C331.2, C331.11 and C427.215 specific for epitopes within HPV16 E6_71-95_ and E6_127-158_ as described in the results section were generated from PBMC of HPV16^+^ cervical cancer patients [30]. In a 96-well plate, HLA-matched DCs were loaded with the indicated reagents in titrated doses. After 24 hours, the DCs were washed and 50,000 T cells were added to each well. After 48 hours, supernatants were taken and subsequently the T cells were cultured in the presence of ^3^H thymidine, which was incorporated in proliferating cells. The level of incorporated ^3^H-thymidine was measured as counts per minute (cpm) after 16 hours on a Liquid Scintillation counter (Wallac). The concentration of IFNγ (in pg/ml) in the supernatants was determined by ELISA (Sanquin).

### *Ex vivo* stimulation of lymph node-derived T cells

PBMC from patient C427 were seeded in 24-wells culture plates at a concentration of 2×10^6^ cells/ml and incubated for 2 hours in X-VIVO15 medium (Lonza), allowing monocytes to adhere to the bottom of the culture wells. After washing thoroughly to remove non-adherent cells, 1 μM of the indicated reagents was loaded on the monocytes for 24 hours. The next day, the monocytes were washed again and then 500,000 LN-derived T cells (LN T cells) of patient C427 were added. During a 3-week culture period, fresh medium was added to the cultures or the cells were divided into new wells when necessary. Autologous monocytes were generated at the end of the culture period and loaded with either 22-mer SLPs [8] covering the amino acid sequences of the conjugated SLPs or with medium (non-stimulated control). The LN T cells were harvested and added to the peptide-loaded or non-loaded monocytes and incubated for 24 hours in the presence of 10 μg/ml Brefeldin A (Sigma-Aldrich) and 10^5^ IU/ml IFNα (Roferon-A; Roche) [32]. The next day, the cells were harvested and stained with fluorescently labeled antibodies specific for CD3, CD4, CD8, CD137, CD154, IFNγ and IL-2 in PBS containing 0.1% saponine, 0.5 % BSA and 0.02% sodium azide. The expression of these markers was analyzed by flow cytometry (BD LSR2).

LN cells derived from an additional 6 cervical cancer patients who had been enrolled in the CIRCLE study (C822, C830, C879, C894, C896, C972) [30] were stimulated with a mix of both AV-SLP conjugates, a mix of both non-conjugated (free) SLPs or medium only. A culture period of 12 days was followed by overnight stimulation with 22-mer peptide loaded (or unloaded for unstimulated control cells) autologous monocytes as described above. For all cultures, a Th1/Th2 cytometric bead array (CBA; BD Biosciences) was performed on supernatant collected from LN cultures on day 7, except for the culture of C879 (collected on day 8). An intracellular cytokine staining was performed to analyze T cell responses after 12 days of culture. Responses with a stimulation index > 3 over non-stimulated cells were considered positive.

### Statistical methods

Statistical significance (Figure 4) was calculated using the Mann-Whitney U test. Statistical significance was defined as p < 0.05. In Table 2, stimulation indices are shown, as calculated by division of the measured cytokine levels over non-stimulated control samples of the same patient. Positive responses defined as SI > 3, shown in bold; < 1 indicates sample value was lower than medium control.

## SUPPLEMENTARY MATERIAL FIGURES


