# Supplementary material for: TLR2 ligand-synthetic long peptide conjugates effectively stimulate tumor-draining lymph node T cells of cervical cancer patients
Source: Oncotarget. 2016 Aug 23;7(41):67087–100. doi: 10.18632/oncotarget.11512 (PMC5341859; doi:10.18632/oncotarget.11512)
Supplement: Supplementary file 1 [file oncotarget-07-67087-s001.pdf]

TLR2 ligand-synthetic long peptide conjugates effectively stimulate tumor-draining lymph node T cells of cervical cancer patients

SUPPLEMENTARY FIGURES

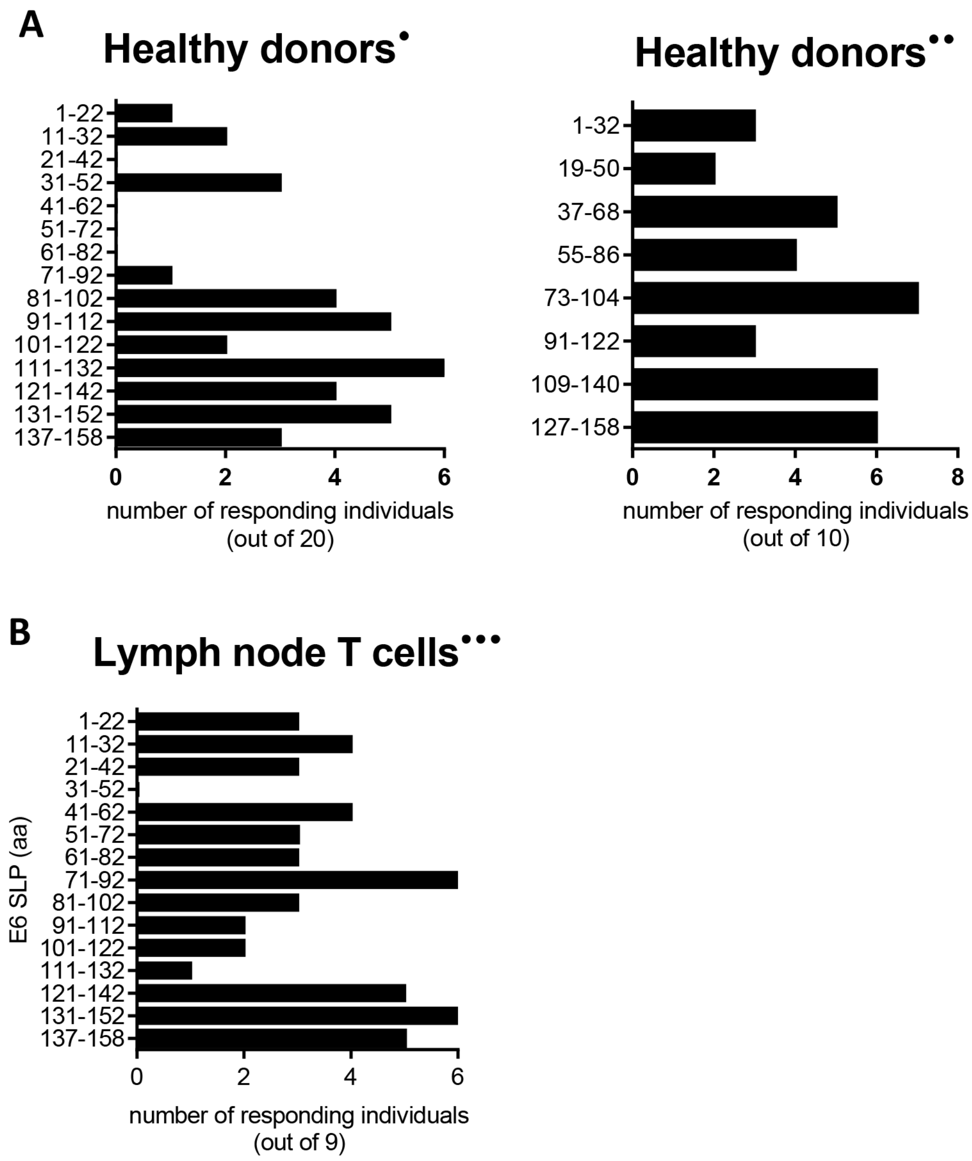

Supplementary Figure S1: Determination of most immunogenic SLPs within HPV16 E6 protein. A. Number of healthy donors with a T cell response against the indicated SLPs overlapping the sequence of HPV16 E6, as measured in a study by (•) Welters et al [22] and (••) De Jong et al [23]. **B.** Number of cervical patients with a LN-derived T cell response against the indicated 22-mers overlapping the sequence of HPV16 E6, as measured in a study by (•••) Vos van Steenwijk et al [3] and unpublished data.

A

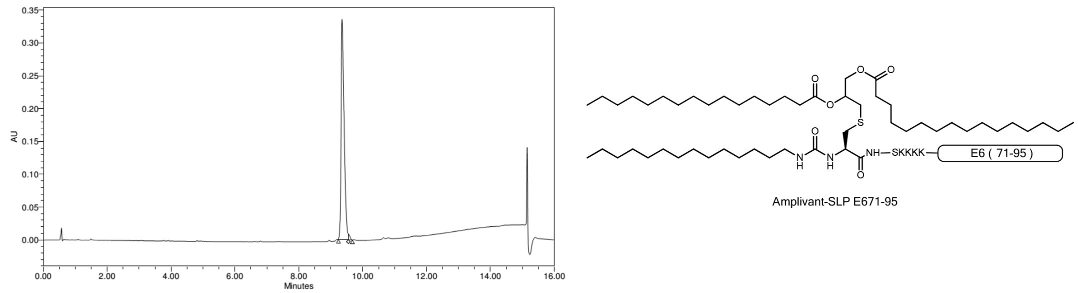

	RT	Area	Height	% Area
1	9.350	2198950	335519	98.7
2	9.580	29581	7361	1.3

B

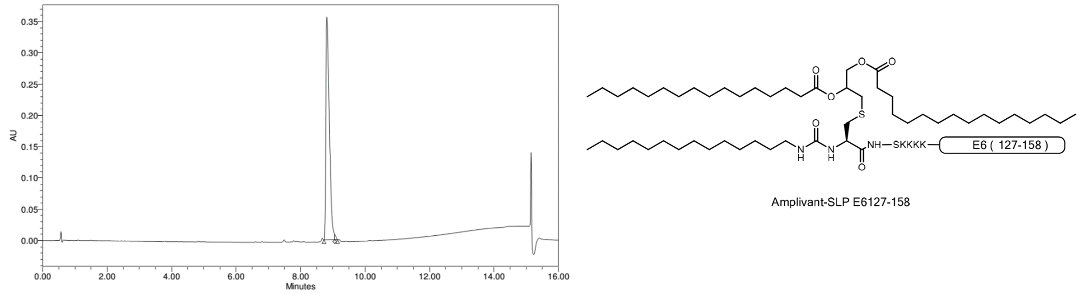

	RT	Area	Height	% Area
1	8.812	2687965	356182	99.3
2	9.086	20084	5587	0.7

Supplementary Figure S2: UV spectrum analysis of clinical GMP-grade TLR2-L SLP conjugates. A. AV-SLP$_{71\text{-}95}$ and **B.** AV-SLP$_{127\text{-}158}$.

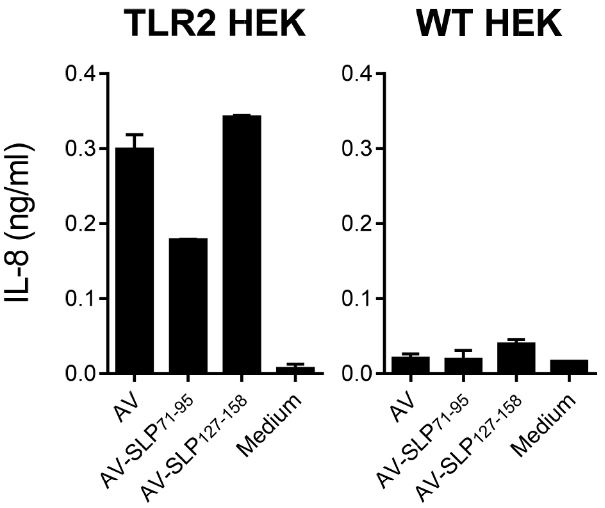

Supplementary Figure S3: Activation of HEK293 cells either transfected with human TLR2 or non-transfected control HEK293 cells. Activation is assessed by measuring the production of IL-8 (ELISA) in the supernatant of cells stimulated with indicated compounds. Error bars represent standard deviation of triplicates.

A

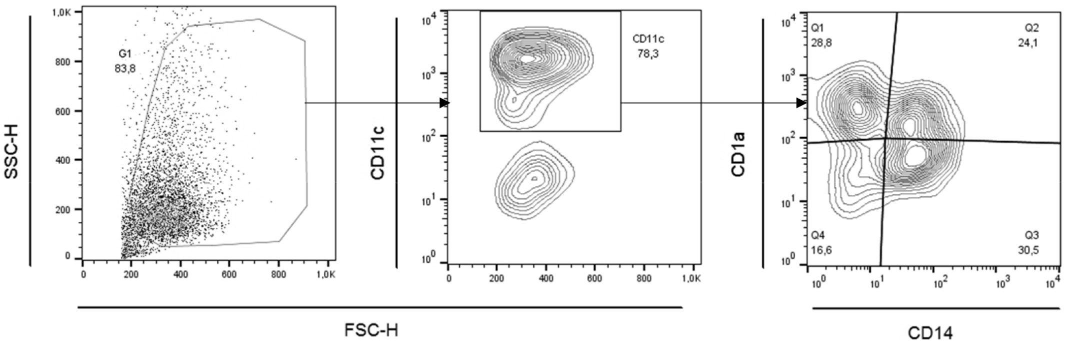

B

Donor 1	CD1a$^+$ (%)	CD1a$^+$CD14$^+$ (%)	CD14$^+$ (%)
**Saline**	50.1	17.7	39.6
**SLPs**	61.9	10.0	22.4
**AV-SLPs**	47.4	21.8	52.3

Donor 2	CD1a$^+$ (%)	CD1a$^+$CD14$^+$ (%)	CD14$^+$ (%)
**Saline**	36.3	25.9	52.2
**SLPs**	41.2	28.9	54.7
**AV-SLPs**	49.4	22	39.6

Donor 3	CD1a$^+$ (%)	CD1a$^+$CD14$^+$ (%)	CD14$^+$ (%)
**Saline**	55.8	26.7	50.6
**SLPs**	69.3	11.8	24.4
**Mixture**	68.7	15.2	31.9
**AV-SLPs**	64.6	23.8	42.7

Donor 4	CD1a$^+$ (%)	CD1a$^+$CD14$^+$ (%)	CD14$^+$ (%)
**Saline**	52.9	24.1	54.6
**SLPs**	46.3	19.6	46.9
**Mixture**	55.1	19.4	41.5
**AV-SLPs**	60.8	20.7	40

Supplementary Figure S4: Skin explant DC migration characteristics. A. Example of flow cytometry gating strategy for skin-migratory cells from human skin explants. Migratory DC were gated based on forward and side scatter (first plot), and on positive CD11c expression (middle plot), and further gated for the different subsets based on CD1a and/or CD14 expression. **B.** Percentages of CD14 and/or CD1a expressing migratory DC subsets within the CD11c$^+$ DC gate.

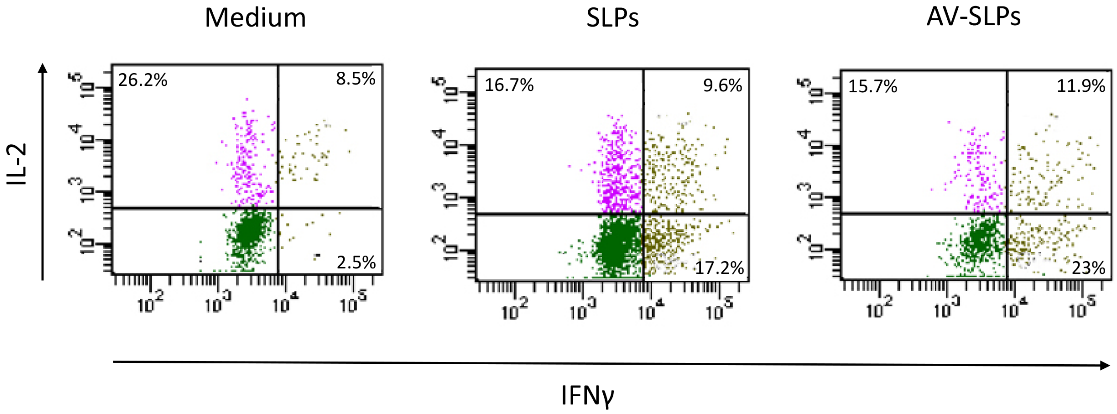

Supplementary Figure S5: Activation of tumor draining LN-derived cells from patient C972 after stimulation with either a mix of SLP$_{71\text{-}95}$ and SLP$_{127\text{-}158}$, a mix of AV-SLP$_{71\text{-}95}$ and AV-SLP$_{127\text{-}158}$ or cultured in medium. The flow cytometry plots depict the intracellular expression of IL-2 and IFNγ by CD4$^+$ CD154$^+$ T cells on day 12 of culture. Numbers in the corner represent the percentages of events in the designated quadrant.
